# Performance of Commercial Mycoplasma hyopneumoniae Serum Enzyme-Linked Immunosorbent Assays under Experimental and Field Conditions

**DOI:** 10.1128/JCM.00485-20

**Published:** 2020-11-18

**Authors:** Ana Paula S. Poeta Silva, Ronaldo L. Magtoto, Henrique M. Souza Almeida, Aric McDaniel, Precy D. Magtoto, Rachel J. Derscheid, Maria M. Merodio, Franco S. Matias Ferreyra, Igor R. H. Gatto, David H. Baum, Maria J. Clavijo, Bailey L. Arruda, Jeffrey J. Zimmerman, Luis G. Giménez-Lirola

**Affiliations:** a Department of Veterinary Diagnostic and Production Animal Medicine, Iowa State University, Ames, Iowa, USA; b Universidade Estadual de São Paulo, Jaboticabal, São Paulo, Brazil; c Pampanga State Agricultural University, Pampanga, Philippines; d PIC North America, Hendersonville, Tennessee, USA; University of Tennessee at Knoxville

**Keywords:** Mycoplasma hyopneumoniae, ELISA, oral fluids, cross-reactivity, test performance, misclassification error rate, *Mycoplasma flocculare*, *Mycoplasma hyopneumoniae*, *Mycoplasma hyorhinis*, *Mycoplasma hyosynoviae*

## Abstract

Mycoplasma hyopneumoniae is an economically significant pathogen of swine. M. hyopneumoniae serum antibody detection via commercial enzyme-linked immunosorbent assays (ELISAs) is widely used for routine surveillance in commercial swine production systems. Samples from two studies were used to evaluate assay performance. In study 1, 6 commercial M. hyopneumoniae ELISAs were compared using serum samples from 8-week-old cesarean-derived, colostrum-deprived (CDCD) pigs allocated to the following 5 inoculation groups of 10 pigs each: (i) negative control, (ii) Mycoplasma flocculare (strain 27399), (iii) Mycoplasma hyorhinis (strain 38983), (iv) Mycoplasma hyosynoviae (strain 34428), and (v) M. hyopneumoniae (strain 232). Weekly serum and daily oral fluid samples were collected through 56 days postinoculation (dpi). The true status of pigs was established by PCR testing on oral fluids samples over the course of the observation period. Analysis of ELISA performance at various cutoffs found that the manufacturers’ recommended cutoffs were diagnostically specific, i.e., produced no false positives, with the exceptions of 2 ELISAs. An analysis based on overall misclassification error rates found that 4 ELISAs performed similarly, although one assay produced more false positives. In study 2, the 3 best-performing ELISAs from study 1 were compared using serum samples generated under field conditions. Ten 8-week-old pigs were intratracheally inoculated with M. hyopneumoniae. Matched serum and tracheal samples (to establish the true pig M. hyopneumoniae status) were collected at 7- to 14-day intervals through 98 dpi. Analyses of sensitivity and specificity showed similar performance among these 3 ELISAs. Overall, this study provides an assessment of the performance of current M. hyopneumoniae ELISAs and an understanding of their use in surveillance.

## Abstract (precis)

Mycoplasma hyopneumoniae is an economically significant pathogen of swine. M. hyopneumoniae serum antibody detection via commercial enzyme-linked immunosorbent assays (ELISAs) is widely used for routine surveillance in commercial swine production systems. Samples from two studies were used to evaluate assay performance. In study 1, 6 commercial M. hyopneumoniae ELISAs were compared using serum samples from 8-week-old cesarean-derived, colostrum-deprived (CDCD) pigs allocated to the following 5 inoculation groups of 10 pigs each: (i) negative control, (ii) Mycoplasma flocculare (strain 27399), (iii) Mycoplasma hyorhinis (strain 38983), (iv) Mycoplasma hyosynoviae (strain 34428), and (v) M. hyopneumoniae (strain 232).

## INTRODUCTION

Mycoplasma hyopneumoniae is an agent in the porcine respiratory disease complex and the cause of enzootic pneumonia in pigs. M. hyopneumoniae colonization of the respiratory cilia can result in suppurative bronchiolitis and lymphoplasmacytic peribronchiolitis and suppression of the immune defenses afforded by the pulmonary mucociliary apparatus, thereby creating an environment in which other pathogens can proliferate and induce more-severe respiratory disease (1). Studies have demonstrated that M. hyopneumoniae infection combined with other bacterial or viral agents, Actinobacillus pleuropneumoniae (2), porcine reproductive and respiratory virus (PRRSV) (3), porcine circovirus type 2 (PCV2) (4), and swine influenza A virus (SIAV) (5), can exacerbate clinical respiratory signs. In addition, M. hyopneumoniae infection and coinfections significantly affect the productivity and profitability of swine production systems. Maes et al. (6) attributed the primary costs of M. hyopneumoniae to antimicrobial treatments used to control secondary infections enabled by M. hyopneumoniae and the significant reduction in growth performance. Holtkamp (https://www.pig333.com/articles/economic-impact-of-mycoplasma-hyopneumoniae-on-pig-farms_8936/) estimated the economic burden of M. hyopneumoniae to the pork industry to be ∼$400 million per year. While disease caused by M. hyopneumoniae is manageable using medication and vaccination, M. hyopneumoniae elimination programs have become more frequent due to their high chances of success (7). Moreover, elimination has been shown to produce a significant return on investment through greater pig productivity and profitability (8).

Successful elimination of and ongoing freedom from M. hyopneumoniae rely on continuous herd monitoring of herd status and testing of replacement animals. Various sampling techniques and diagnostic tools have been developed to detect either M. hyopneumoniae organisms, antigens, or antibodies (9). M. hyopneumoniae organisms can be isolated using culture; however, routine M. hyopneumoniae isolation poses challenges due to the low growth rate and requirement for specific media (10–12). The use of quantitative PCR testing for M. hyopneumoniae DNA has increased and, theoretically, should offer the highest likelihood of detection at early stages of infection (13). Although detection of subclinically infected pigs harboring low levels of the pathogen may support M. hyopneumoniae control and prevention programs, the potential environmental contamination of samples with the environment can undermine confidence in the process (14).

M. hyopneumoniae antibody detection is the most common and economical approach to M. hyopneumoniae surveillance (9), but its use requires a thorough understanding of test performance in the context of the specific testing objective(s). In the field, early detection of M. hyopneumoniae infections is a challenge, but diagnostic accuracy is likewise an issue for routine surveillance. Furthermore, over the course of a successful elimination effort and as the M. hyopneumoniae-negative proportion of the population increases, diagnostic specificity (false positives) becomes increasingly important. Given that various commercial M. hyopneumoniae enzyme-linked immunosorbent assays (ELISAs) are available on the market, the objective of this study was to compare their performance under experimental and field conditions.

## MATERIALS AND METHODS

### Experimental design.

**(i) Study 1.** Six commercial Mycoplasma hyopneumoniae ELISAs were evaluated using serum samples (*n* = 680) derived from an experiment previously conducted at Iowa State University (ISU) under the supervision of the Institutional Office for Responsible Research (15). In brief, samples were collected over the course of 59 days from 8-week-old cesarean-derived, colostrum-deprived (CDCD) pigs in 5 inoculation groups: (i) Friis media (negative control; *n* = 10), (ii) Mycoplasma flocculare (*n* = 10), (iii) Mycoplasma hyorhinis (*n* = 10), (iv) Mycoplasma hyosynoviae (*n* = 10), and (v) Mycoplasma hyopneumoniae (*n* = 10). Longitudinal sampling of group M. hyopneumoniae allowed for the temporal evaluation of antibody detection, and samples from non-M. hyopneumoniae groups permitted the evaluation of M. hyopneumoniae ELISAs for nonspecific reactions. At the termination of the experiment, all pigs were humanely euthanized by penetrating captive bolt followed by exsanguination. Lung tissues collected at necropsy from group M. hyopneumoniae were tested by PCR to verify M. hyopneumoniae infection. The performance of six commercial ELISAs was evaluated at various cutoffs and scored in terms of misclassification error rate, i.e., indices of false positives and false negatives.

**(ii) Study 2.** Three commercial M. hyopneumoniae ELISAs from study 1 were further evaluated using serum samples (*n* = 362) collected over the course of an M. hyopneumoniae field study. Briefly, 10 8-week-old animals in one pen (inoculated pen) in a room housing 1,250 pigs in 46 pens (∼28 pigs per pen) were intratracheally administered an M. hyopneumoniae inoculum. Thereafter, paired individual serum and tracheal samples were collected from one noninoculated pig in each of the 46 pens at 7- to 14-day intervals for 98 days. At the termination of the study, samples were tested for DNA (tracheal samples) and antibody (serum). The performance of the three commercial ELISAs was evaluated using a logistic mixed regression.

### Studies.

**(i) Study 1.** Animals were allocated to 5 rooms (treatments) with 5 pens per room and 2 pigs per pen. Prior to inoculation, they were determined to be free of mycoplasmal infections on the basis of mycoplasma-specific ELISA and quantitative real-time PCR (qPCR) testing of serum, oral fluid, or tonsil scraping (M. hyosynoviae group only) samples (15).

Pigs were inoculated as described below in Table 1. Mycoplasma inocula were created from specimens from the Mycoplasma Laboratory in the Veterinary Diagnostic Laboratory at Iowa State University (VDL-ISU). Negative-control animals were inoculated intranasally with Friis media (1 ml per naris). To reduce the stress associated with inoculation (<3-min restraint procedure), pigs in M. flocculare, M. hyorhinis, and M. hyosynoviae groups were sedated by intramuscular administration of a solution containing a combination of tiletamine hydrochloride and zolazepam hydrochloride (5 mg; Telazol, Zoetis, Parsippany, NJ, USA), xylazine (250 mg; MilliporeSigma, St. Louis, MO, USA), and ketamine (250 mg; Merial, France) at a dose of 1 ml per 4.4 kg of body weight.

**TABLE 1:** *Mycoplasma* inoculation groups (10 pigs per group)^*a*^

Study	Inoculum	Route of inoculation (dose [ml])	Concn^*b*^
1	Negative control (Friis media)	Intranasal (0.5/nostril)	NA
1	*M. flocculare* strain 27399	Tonsil of the soft palate (2)	1.0 × 10^5^ CCU/ml
		Intranasal (1)	1.0 × 10^5^ CCU/ml
		Intratracheal (1)	1.0 × 10^5^ CCU/ml
1	*M. hyorhinis* strain 38983	Tonsil of the soft palate (2)	3.2 × 10^8^ CFU/ml
		Intraperitoneal (2)	3.2 × 10^8^ CFU/ml
1	*M. hyosynoviae* strain 34428	Tonsil of the soft palate (2)	2.1 × 10^9^ CFU/ml
		Intranasal (0.5/nostril)	2.1 × 10^9^ CFU/ml
		Intravascular (1 ml/ear vein)	2.1 × 10^9^ CFU/ml
1	M. hyopneumoniae strain 232^*c*^	Intratracheal (1 ml)	1.0 × 10^6^ CCU/ml
2		Intratracheal (10 ml)	1.0 × 10^5^ CCU/ml

a Table adapted from reference 14.

b The purity of original seeds and final inoculum was evaluated by qPCR and microscopy staining (×1,000 magnification) to rule out bacterial contamination, including other *Mycoplasma* spp.

c M. hyopneumoniae strain 232 inoculum was lung tissue from an infected pig homogenized and diluted 1:100 in Friis media.

M. hyopneumoniae (strain 232) inoculum consisted of lung tissue homogenate (lot 43; Veterinary Diagnostic Laboratory, ISU) from M. hyopneumoniae-inoculated CDCD pigs diluted in 1:100 of a modified Friis broth media (9) to a concentration estimated at 1.0 × 10^6^ color-changing units (CCU)/ml by titration in Friis broth media (10–12). The diluted inoculum was administered intratracheally (10 ml) to pigs in the M. hyopneumoniae group as previously documented (16).

M. flocculare (strain 27399) inoculum consisted of a live culture propagated in Friis broth media in a shaking water bath (37°C for 48 h) diluted to a concentration at 1.0 × 10^5^ CCU/ml, as determined by titration in Friis media. The inoculum was administered intranasally (1 ml in each nostril) and intratracheally (1 ml in the trachea). In addition, on 0 and 4 days postinoculation (dpi), the inoculum was “scrubbed” on the tonsils of the soft palate using a 16-in. (41-cm) large-tip cotton swab (Scopettes 16-in. absorbent; Birchwood Laboratories, Inc., Eden Prairie, MN, USA) saturated with 2 ml of the inoculum.

M. hyorhinis (strain 38983) inoculum consisted of a live culture propagated in Friis broth media (37°C for 48 h) and diluted to a concentration of 3.2 × 10^8^ CCU/ml, as determined by titration in Friis agar media. The inoculum was administered into the peritoneal cavity (2 ml) and “scrubbed” on the tonsil of the soft palate using a 16-in. (41-cm) large-tip cotton swab (Scopettes 16-in. absorbent) saturated with 2 ml of the inoculum.

M. hyosynoviae (strain 34428) field isolate was cultivated in Difco medium containing turkey serum (d-TS) broth (37°C for 48 h) and diluted to a concentration of 2.1 × 10^9^ CCU/ml, as determined by titration in Friis agar media. Pigs were inoculated intranasally (1 ml per naris) and intravenously (1 ml via the auricular vein) and “scrubbed” on the tonsils of the soft palate using a 16-in. (41 cm) large-tip cotton swab (Scopettes16-in. absorbent) saturated with 2 ml of the inoculum.

**(ii) Study 2.** The M. hyopneumoniae (strain 232) inoculum used in study 2 was identical to that in study 1 except that the concentration was estimated to be 1 × 10^5^ CCU/ml, and each of the 10 M. hyopneumoniae-inoculated pigs was intratracheally administered 10 ml.

### Sample collection.

Blood samples were collected using single-use collection tubes. In the laboratory, samples were centrifuged (1,500 × *g* for 15 min), and the serum was aliquoted into 2 ml cryogenic tubes (Cryo.s; Greiner Bio-One, Monroe, NC, USA) for storage at −80°C.

Pen-based oral fluid samples were collected using 3-strand (1.6 cm) 100% cotton rope (Web Rigging Supply, Lake Barrington, IL, USA) suspended from a bracket fixed to the side of the enclosure (17). The oral fluid sample was recovered from the rope after 30 min exposure to the pigs. In the laboratory, oral fluid samples were vortexed and aliquoted into cryogenic tubes (Cryo.s) and stored at −80°C.

Tracheal mucosal samples were collected using a sheathed, single-use porcine cervical artificial insemination catheter (Nasco, Fort Atkinson, WI, USA). After sampling, the tip containing the collected material was severed from the catheter and placed in a tube containing 1 ml sterile phosphate-buffered saline (PBS). In the laboratory, tracheal samples were transferred to 2 ml cryogenic tubes (Cryo.s) for storage at −80°C.

In study 1, Blood samples were collected from all pigs on −3, 0, 3, 7, 10, 14, 17, 21, 24, 28, 35, 42, 49, and 56 dpi, and pen-based oral fluid samples were collected daily. Lung tissues were collected at necropsy (56 dpi) and stored at −80°C until processed for PCR testing.

In study 2, In the inoculated pen, tracheal samples (0, 15, and 28 dpi) and blood samples (0, 15, 28, 42, and 56 dpi) were collected from the 10 M. hyopneumoniae-inoculated pigs and 1 noninoculated pig. In the remaining pens, tracheal and blood samples were collected from one pig on 15, 24, 42, 56, 71, 85, and 98 dpi.

### M. hyopneumoniae ELISAs.

Six commercial M. hyopneumoniae serum antibody ELISAs were included in the comparison (Table 2). Three assays were indirect ELISAs based on the detection of anti-P46 antibodies (SK108 Mhyo, BioChek, Berkshire, UK; *M. hyo* Ab test, IDEXX Laboratories Inc., Westbrook, ME, USA; and ID Screen Mycoplasma hyopneumoniae Indirect, IDvet, Grabels, France). Two assays were blocking ELISAs based on the detection of antibody against a conserved epitope of the M. hyopneumoniae 74-kDa protein (INgezim *M. hyo* Compac, Eurofins Ingenasa, Madrid, Spain; IDEIA Mycoplasma hyopneumoniae EIA kit, Oxoid Limited, Hampshire, UK). Distinct from the other ELISAs, one assay (Civtest Suis Mhyo, Laboratorios Hipra, S.A., Girona, Spain) was based on a two-well indirect ELISA format. As described by the manufacturer, one well contained an M. hyopneumoniae-specific antigen and the other contained generic mycoplasma antigen. The response was then determined as the proportion of M. hyopneumoniae-specific antibodies in the sample.

**TABLE 2:** Overview of commercial Mycoplasma hyopneumoniae ELISAs evaluated

ELISA (format)^*a*^	Incubation (time [min], temp [°C])^*b*^	Standardized response^*c*^	Response interpretations
1 (indirect)	1: 30, 22–27; 2: 30, 22–27; 3: 15, 22–27	$\text{S/P}={\text{TS}}_{\text{OD}}-{\text{NC}}_{\text{OD}}\text{/}{\text{PC}}_{\text{OD}}-{\text{NC}}_{\text{OD}}$	S/P < 0.50 = negative; S/P ≥ 0.50 = positive
2 (blocking)	1: 60, 18–25; 2: 30, 18–25; 3: 10, 22–27	$\%\text{B}={\text{TS}}_{\text{OD}}\times 100\text{/}{\text{NC}}_{\text{OD}}$	%B ≤ 55% = negative; 55% > %B < 60% = suspect; %B ≥ 60% = positive
3 (indirect)	1: 30, 18–26; 2: 30, 18–26; 3: 15, 18–26	$\text{S/P}={\text{TS}}_{\text{OD}}-{\text{NC}}_{\text{OD}}\text{/}{\text{PC}}_{\text{OD}}-{\text{NC}}_{\text{OD}}$	S/P < 0.3 = negative; 0.3 ≤ S/P ≥ 0.4 = suspect; S/P > 0.4 = positive
4 (indirect)	1: 45, 34–40; 2: 30, 34–40; 3: 15, 16–26	$\text{S/P}={\text{TS}}_{\text{OD}}-{\text{NC}}_{\text{OD}}\text{/}{\text{PC}}_{\text{OD}}-{\text{NC}}_{\text{OD}}$	S/P < 0.3 = negative; 0.3 ≤ S/P ≥ 0.4 = suspect; S/P > 0.4 = positive
5 (indirect)	1: 60, 36–38; 2: 60, 36–38; 3: 15, 20–25	$\text{RI}=100\times \left({\text{TS}}_{\text{OD}}-{\text{NC}}_{\text{OD}}\text{/}{\text{PC}}_{\text{OD}}-{\text{NC}}_{\text{OD}}\right)$	RI < 30 = negative; 30 ≤ RI ≥ 35 = suspect; RI > 35 = positive
6 (blocking)	1: 90, 20–30; 2: 15, 20–30; 3: 10, 20–30	$\%\text{B}=\text{mean }{\text{TS}}_{\text{OD}}$	%B ≥ 65% = negative; 50% ≤ %B > 65% = suspect; %B < 50% = positive

a ELISA 1, SK108 Mhyo, BioChek, Berkshire, UK; ELISA 2, INgezim *M. hyo* Compac, Eurofins Ingenasa, Madrid, Spain; ELISA 3, *M. hyo* Ab test, IDEXX Laboratories Inc.; ELISA 4, ID Screen Mycoplasma hyopneumoniae Indirect, IDvet, Grabels, France; ELISA 5, Civtest Suis Mhyo, Laboratorios Hipra, S.A., Girona, Spain; ELISA 6, IDEIA Mycoplasma hyopneumoniae EIA kit, Oxoid Limited, Hampshire, UK.

b Incubation conditions for specific steps in the ELISA procedure are as follows: 1, after adding sample; 2, after adding conjugate reagent; and 3, after adding substrate reagent. In addition, BioChek ELISA mandates an incubation of 30 min at 22 to 27°C after adding stop solution.

c TS, test samples, NC, negative control; PC, positive control; OD, optical density.

In study 1, we tested 680 serum samples from the 5 inoculation groups on each of the six commercial M. hyopneumoniae ELISAs: (i) M. hyorhinis (*n* = 129), (ii) M. hyosynoviae (*n* = 131), (iii) M. flocculare (*n* = 140), (iv) M. hyopneumoniae (*n* = 140), and (v) negative control (*n* = 140). To avoid bias, each sample was randomly assigned to a specific ELISA plate and well (R program version 3.6.0; R Core Team 2019, Vienna, Austria).

In study 2, we tested 362 serum samples from M. hyopneumoniae-inoculated (*n* = 44) and noninoculated pigs (*n* = 318) on three commercial M. hyopneumoniae ELISAs (SK108 Mhyo, *M. hyo* Ab test, and Civtest Suis Mhyo).

The same ELISA equipment, i.e., plate washer (ELx405; Biotek Instruments Inc., Winooski, VT USA), ELISA reader (EMax Plus microplate reader; Molecular Devices, San Jose, CA USA), and reader software (SoftMax Pro 7.0; Molecular Devices) were used throughout. One commercial M. hyopneumoniae ELISA was run per day following the instructions provided by the manufacturer. Assay validity criteria were checked and verified for each plate according to the instructions provided by each manufacturer. Serum samples were randomized to avoid systematic errors, and no samples were retested.

### Detection of M. flocculare, M. hyorhinis, and M. hyosynoviae DNA and antibodies.

In study 1, as described elsewhere (15), oral fluid and serum samples were tested for *Mycoplasma* species-specific DNA and antibodies initially to confirm pigs’ negative status and later to corroborate M. flocculare, M. hyorhinis, and M. hyosynoviae infections in inoculated animals (0 to 56 dpi). *Mycoplasma* DNA extraction was performed using a commercial kit (MagMax-96 Pathogen RNA/DNA kit (Applied Biosystems, Carlsbad, CA USA) on the Kingfisher Flex system (Thermo Fisher Scientific, Waltham, MA, USA) and amplified on Applied Biosystems 7500 real-time PCR (Thermo Fisher Scientific). Antibody responses against M. flocculare and M. hyosynoviae were assessed using Tween 20-extracted surface protein-based ELISAs. M. hyorhinis-specific antibody testing used a chimeric VlpA to VlpG recombinant protein-based ELISA (15).

### Detection of M. hyopneumoniae DNA in oral fluids.

In study 1, daily oral fluid samples were tested for M. hyopneumoniae DNA to monitor the infection in M. hyopneumoniae-inoculated animals. Weekly oral fluid samples (0, 7, 14, 21, 28, 35, and 42 dpi) from M. flocculare, M. hyorhinis, M. hyosynoviae, and negative control groups were tested for M. hyopneumoniae DNA to establish that they remained free of the pathogen. In total, 332 oral fluid samples from the following 5 inoculation groups were tested for M. hyopneumoniae DNA: (i) M. hyorhinis (*n* = 33), (ii) M. hyosynoviae (*n* = 39), (iii) M. flocculare (*n* = 44), (iv) M. hyopneumoniae (*n* = 276), and (v) negative control (*n* = 36).

M. hyopneumoniae DNA detection was based on commercial kits (RealPCR DNA/RNA magnetic bead kit, RealPCR master mix, RealPCR *M. hyo* DNA mix; IDEXX) performed as directed by the manufacturer. DNA was extracted on the Kingfisher Flex system and amplified on Applied Biosystems 7500 real-time PCR. Each plate included a known M. hyopneumoniae-positive sample and a negative control (RNA-free water). A test result was considered valid when the internal positive cycle threshold (*C_T_*) values were ≤36. A sample was considered M. hyopneumoniae positive when *C_T_* values were ≤40.

### Detection of M. hyopneumoniae DNA in tracheal samples and lung homogenates.

Lung tissues were collected at necropsy (56 dpi) and stored at −80°C until they were processed for PCR testing. As described elsewhere (18), lung (3 by 3 cm) containing both normal and affected tissue was minced using sterile scissors and then placed in a 50-ml conical tube with 30 ml of Earle’s balanced salt solution (Sigma-Aldrich, St. Louis, MO, USA) at a concentration of 10% (wt/vol). The sample was homogenized (2 min at 1,000 rpm; Geno/Grinder; Spex SamplePrep, Metuchen, NJ, USA) and then centrifuged (10 min at 4,200 × *g*).

M. hyopneumoniae DNA detection for both tracheal and lung homogenates was based on commercial kits (MagMax-96 Pathogen RNA/DNA kit, PCR VetMax-Plus qPCR master mix, VetMax Mycoplasma hyopneumoniae reagents; Applied Biosystems) performed as directed by the manufacturer. DNA was extracted on the Kingfisher Flex system and amplified on Applied Biosystems 7500 real-time PCR. Each plate included a known M. hyopneumoniae-positive sample (VetMax-Plus qPCR master mix kit includes Xeno DNA Control, Applied Biosystems) and a negative control sample (RNA-free water). A test result was considered valid when the internal positive *C_T_* value was ≤36. A sample was considered M. hyopneumoniae positive when *C_T_* values were ≤37.

### Data analysis.

**(i) Study 1.** Serum antibody data from 680 samples were identified by inoculation group (negative control, M. flocculare, M. hyorhinis, M. hyosynoviae, and M. hyopneumoniae), pig number (1 to 50), and the standardized response for each of the six ELISAs, i.e., sample-to-positive ratio (S/P), percent blocking (%B), or relative index (RI). Evaluation of the six ELISA results revealed that the data were not normally distributed (Shapiro-Wilk W test; *P < *0.05). Transformation using logarithms or the Box-Cox method was not successful but was achieved using “ordered quantile normalization” (19). Ordered quantile normalization was performed using the function orderNorm of the bestNormalize package (20) in R (R version 3.6.0, R Core Team 2019) for each of the six sets of ELISA results. Transformed ELISA results were normally distributed, i.e., lain on a straight line in quantile-quantile plots, and the Shapiro-Wilk W test was not significant (*P > *0.05).

The evaluation of test performance was constrained by the small number of M. hyopneumoniae-infected animals (*n* = 8 pigs) and the delayed antibody response in these pigs. Initially, test results from non-M. hyopneumoniae groups (*n* = 40 pigs) were used to evaluate the impact of alternative cutoffs on the false-positive rate for each manufacturer’s assay. As described elsewhere (21), alternative cutoffs for BioChek, Eurofins Ingenasa, IDEXX, IDvet, and Hipra ELISAs were established as the mean of the results from non-M. hyopneumoniae groups plus two or three standard deviations (*x̄* + 2, 3 SD), i.e., the values localized at the 97.5% and 99.7% quantiles of the normal distribution (upper tail values), respectively. Because Oxoid ELISA defined positives as results with <50% blocking, the alternative cutoffs were established as (*x̄* − 2 and 3 SDs), i.e., the values located at the 2.5% and 0.3% quantiles of the normal distribution, respectively (lower tail values). Thereafter, these alternative cutoffs were back transformed to the original ELISA units (S/P, %B, or RI), using the predict function in R.

Overall misclassification error rates (false positives and false negatives) for the six commercial M. hyopneumoniae ELISAs were determined by Poisson regression using generalized estimating equations (PROC GENMOD, SAS v.9.4; SAS Institute, Cary, NC). For the misclassification analysis, false-positive results were defined as any positive result from non-M. hyopneumoniae-inoculated pigs or from M. hyopneumoniae-inoculated pigs at <21 dpi (“suspect” results on 14 or 17 dpi were not penalized). False-negative results were defined as negative results from M. hyopneumoniae-inoculated pigs ≥21 dpi. Two pigs in the M. hyopneumoniae group did not become M. hyopneumoniae infected via inoculation or contact, i.e., were free of M. hyopneumoniae infection on the basis of M. hyopneumoniae PCR testing of lung homogenate collected at the termination of the experiment, and were excluded from the misclassification error rate analysis.

The Poisson regression model used the count of misclassification responses as the dependent variable, ELISA as the independent variable, and dpi as a repeated measure, and it assumed a compounded symmetry correlation structure across dpi (22). The model goodness of fit was evaluated by residual deviance testing (chi-square test). The misclassification error rate was calculated as the exponential of beta coefficients estimated by the Poisson regression model and interpreted as the overall incident rate of misclassification errors over time. The analyses were considered statistically significant at *P ≤ *0.05.

**(ii) Study 2.** Serum samples (44 from inoculated pigs, 318 from noninoculated pigs) were tested on BioChek, IDEXX, and Hipra ELISAs. For noninoculated pigs, individual animal M. hyopneumoniae status was established by PCR testing of tracheal samples (positive or negative).

The diagnostic sensitivity and specificity of each of the three commercial M. hyopneumoniae ELISAs were estimated by logistic regression using GEE (PROC GENMOD; SAS v.9.4) with the qualitative ELISA result as the dependent variable, sample status (based on PCR testing of tracheal samples) as the independent variable, and pen as a repeated measure (1 to 46), and assumed a heterogeneous first-order autoregressive covariance-variance structure (22). Model goodness of fit was evaluated by residual deviance testing (chi-square test). Diagnostic sensitivity (or specificity) was estimated by modeling the probability of a positive (negative) ELISA result given a positive (negative) tracheal sample PCR testing result. Receiver operating characteristic (ROC) and area under the curve (AUC) analyses were based on the marginal predicted probabilities from the diagnostic sensitivity models using PROC LOGISTIC (SAS v.9.4).

## RESULTS

### Study 1.

Clinical signs and pathological lesions at necropsy were reported in a previous study (15). In brief, no clinical signs were observed in pigs in the negative control, M. flocculare, or M. hyopneumoniae groups, and no gross lesions were observed at necropsy. In M. hyorhinis-inoculated pigs, mild front and hind limb lameness, swollen joints, rough hair coat, and loss of condition were observed in eight pigs. Two pigs in this group were humanely euthanized at 24 dpi due to anorexia and reluctance to move. Necropsy of these animals revealed polyarthritis and polyserositis. Likewise, in the M. hyosynoviae group, swollen joints (hocks) were observed in seven pigs, and increased joint fluid volume was observed at necropsy. One M. hyosynoviae-inoculated pig died during blood collection on 10 dpi.

M. hyopneumoniae DNA testing of pen-based oral fluid samples collected daily from M. hyopneumoniae-inoculated animals (*n* = 276) confirmed productive M. hyopneumoniae infection in the inoculated group. Overall, 80 of 276 (29.0%) oral fluid samples were positive for M. hyopneumoniae DNA, with the first PCR-positive at 9 dpi. At the individual pig level, necropsy and DNA testing of individual pig lung homogenates revealed that 8 of 10 pigs had been infected, i.e., 2 pigs showed no gross lesions compatible with M. hyopneumoniae, were M. hyopneumoniae DNA negative, and showed no evidence of an antibody response against M. hyopneumoniae. Therefore, these 2 pigs were removed from the study, and, for this reason, the statistical analyses were based on 8 M. hyopneumoniae-infected pigs. M. hyopneumoniae DNA testing of weekly pen-based oral fluid samples from M. flocculare (n = 44), M. hyorhinis (n = 33), M. hyosynoviae (*n* = 39), and negative control groups (*n* = 36) were all negative, i.e., no inadvertent contamination of these pigs or samples from these groups with M. hyopneumoniae occurred during the experiment.

M. hyorhinis DNA was detected in one serum sample (10 dpi) from the M. hyorhinis group at 10 dpi and consistently in pen-based oral fluid samples from 2 dpi through 56 dpi. M. hyosynoviae DNA was detected in two serum samples (3 and 7 dpi) from two M. hyosynoviae-inoculated pigs and intermittently in pen-based oral fluid samples from 4 dpi through 15 dpi. M. flocculare DNA was not detected in serum or oral fluid samples collected during the study. M. hyorhinis and M. hyosynoviae antibody responses were detected at earlier 10 dpi in serum samples. No M. flocculare antibody response was detected in serum during the study. Thus, M. hyorhinis and M. hyosynoviae groups were infected using the inoculation procedures described in Table 1, but there was no evidence of infection in M. flocculare-exposed pigs.

A total of 680 serum samples were collected over the course of the study (−3 to 56 dpi) from the pigs in the 5 treatment groups. For the analysis of the ELISAs, the 540 serum testing results from the non-M. hyopneumoniae inoculated pigs were used to evaluate the false-positive rates in the 6 ELISAs. Four cutoffs were considered: (i) manufacturer's cutoff with suspect considered negative, (ii) manufacturer's cutoff with suspect considered positive, (iii) cutoff based on the mean of the results from non-M. hyopneumoniae groups plus two standard deviations (*x̄* + 2 SD), and (iv) cutoff based on the mean of the results from non-M. hyopneumoniae groups plus three standard deviations (*x̄* + 3 SD).

As shown in Table 3, the manufacturer's recommended cutoffs resulted in zero or few false positives, especially if suspect results were interpreted as negative. Cutoffs calculated as *x̄* ± 2 or 3 SD were generally less stringent than the manufacturer's cutoffs and, with exception of IDvet ELISA, resulted in more false-positive results. No patterns in false-positive responses were observed between any of the ELISAs and non-M. hyopneumoniae-inoculated groups, i.e., negative control, M. flocculare, M. hyorhinis, and M. hyosynoviae.

**TABLE 3:** Mycoplasma hyopneumoniae antibody ELISA false-positive rate as a function of assay cutoff in study 1^*a*^

ELISA^*b*^	Manufacturer's cutoff for suspect as:		Cutoff using mean of negative samples plus:	
	Negative	Positive	2 SDs	3 SDs
1^*c*^^,^^*f*^	S/P ≥ 0.50 (0/540)	S/P ≥ 0.50 (0/540)	S/P ≥ 0.08 (14/540)	S/P ≥ 0.15 (1/540)
2^*d*^	%B ≥ 60% (0/540)	%B > 55% (0/540)	%B > 49% (14/540)	%B > 54.7% (1/540)
3^*c*^	S/P ≥ 0.40 (0/540)	S/P > 0.30 (0/540)	S/P > 0.07 (11/540)	S/P > 0.13 (1/540)
4^*c*^	S/P > 0.40 (4/540)^*g*^	S/P > 0.30 (5/540)^*g*^	S/P > 0.19 (14/540)	S/P > 0.54 (1/540)
5^*e*^	RI > 35 (0/540)	RI ≥ 30 (0/540)	RI > 5.7 (14/540)	RI > 13.7 (1/540)
6^*d*^	%B < 50% (1/540)^*h*^	%B < 65% (14/540)^*h*^	%B < 65% (14/540)	%B < 49.2% (1/540)

a Based on testing 540 serum samples from M. hyopneumoniae-negative pigs, i.e., M. flocculare, M. hyorhinis, M. hyosynoviae, or negative control groups.

b ELISA 1, SK108 Mhyo, BioChek, Berkshire, UK; ELISA 2, INgezim *M. hyo* Compac, Eurofins Ingenasa, Madrid, Spain; ELISA 3, *M. hyo* Ab test, IDEXX Laboratories Inc.; ELISA 4, ID Screen Mycoplasma hyopneumoniae Indirect, IDvet, Grabels, France; ELISA 5, Civtest Suis Mhyo, Laboratorios Hipra, S.A., Girona, Spain; ELISA 6, IDEIA Mycoplasma hyopneumoniae EIA kit, Oxoid Limited, Hampshire, UK.

c Result expressed as sample-to-positive ratio (S/P) (see Table 2).

d Percent blocking (%B) (see Table 2).

e Relative index (RI) (see Table 2).

f This manufacturer does not include a suspect classification (see Table 2).

g False-negative and false-positive results (*n* = 4 and *n* = 5, respectively) were from one pig in the negative control group.

h False-negative results (*n* = 1) were from one pig in the M. hyorhinis group. False-positive results (*n* = 14) were from 2 pigs in negative control group, 3 pigs in M. flocculare group, 2 pigs in M. hyorhinis group, and 3 pigs in M. hyosynoviae group.

The detection of an M. hyopneumoniae antibody over time in the 8 individual pigs colonized by M. hyopneumoniae is shown in Table 4. A detectable antibody was slow to develop, and its appearance was inconsistent among pigs and ELISAs. Because of limitations in the experimental design, i.e., relatively few samples from M. hyopneumoniae antibody-positive pigs, the overall ELISA comparison was based on misclassification errors under two scenarios, i.e., with suspect results interpreted as negative or positive (Table 5). For both cases, no significant difference (*P < *0.05) in the misclassification rate was detected among BioChek, IDEXX, Hipra, and Oxoid ELISAs, whereas significantly higher misclassification rates were observed in Eurofins Ingenasa and IDvet ELISAs. With suspect considered negative, the misclassification error rates for Eurofins Ingenasa and IDvet ELISAs were 2.07 and 3.28, respectively, i.e., 107% and 228% higher than the other ELISA used in the comparison. With suspect considered positive, the misclassification rates for Eurofins Ingenasa and IDvet ELISAs were 2.36 and 3.00, respectively, i.e., 126% and 200% higher than the comparison ELISAs.

**TABLE 4:** Mycoplasma hyopneumoniae antibody detection by day postinoculation in study 1

Pig	ELISA^*a*^	Result on dpi^*b*^:								
		14	17	21	24	28	35	42	49	56
41	1								+	+
	3								+	+
	5								+	+
	6						S	+	+	+
42	1					+	+	+	+	+
	2						+	+	+	+
	3					S	+	+	+	+
	4						S	S	+	+
	5					+	+	+	+	+
	6	S	S	+		+	+	+	+	+
43	1							+	+	+
	3							S	+	+
	4								S	+
	5							+	+	+
	6						+	+	+	+
44	5									S
	6								S	+
46	1					+	+	+	+	+
	2							+	+	+
	3					S	+	+	+	+
	4							S		+
	5					+	+	+	+	+
	6		S	S	+	+	+	+	+	+
47	1					+	+	+	+	+
	2					S	+	+	+	+
	3					+	+	+	+	+
	4						S	+	+	+
	5					+	+	+	+	+
	6	S	S	+	+	+	+	+	+	+
48	1					+	+	+	+	+
	2						S	+	+	+
	3					+	+	+	+	+
	4					+	+	+	+	+
	5				+	+	+	+	+	+
	6		S	+	+	+	+	+	+	+
49	1				+	+	+	+	+	+
	2						+	+	+	
	3				S	+	+	+	+	+
	4						S		+	
	5				+	+	+	+	+	+
	6		S	+	+	+	+	+	+	+

a ELISA 1, SK108 Mhyo, BioChek, Berkshire, UK; ELISA 2, INgezim *M. hyo* Compac, Eurofins Ingenasa, Madrid, Spain; ELISA 3, *M. hyo* Ab test, IDEXX Laboratories Inc.; ELISA 4, ID Screen Mycoplasma hyopneumoniae Indirect, IDvet, Grabels, France; ELISA 5, Civtest Suis Mhyo, Laboratorios Hipra, S.A., Girona, Spain; ELISA 6, IDEIA Mycoplasma hyopneumoniae EIA kit, Oxoid Limited, Hampshire, UK.

b +, antibody positive; S, suspect.

**TABLE 5:** Misclassification errors^*a*^ (count) for six Mycoplasma hyopneumoniae antibody ELISAs by day postinoculation in study 1

Status	ELISA^*b*^	No. with status on dpi:														Total	Misclassification error rate^*c*^
		–3	0	3	7	10	14	17	21	24	28	35	42	49	56		
Suspect assumed negative	1	0	0	0	0	0	0	0	8	8	3	3	2	1	1	26	1.86 (0.86–4.00) A
	2	0	0	0	0	1	1	1	7	6	2	2	2	1	1	24	2.07 (1.01–4.24) B
	3	0	0	0	0	0	0	0	8	8	5	3	3	1	1	29	1.79 (0.84–3.79) A
	4	0	0	0	0	0	0	1	8	9	7	8	6	4	3	46	3.28 (1.88–5.71) C
	5	0	0	0	0	1	1	1	7	7	7	4	2	3	2	35	1.71 (0.90–3.24) A
	6	0	0	0	0	0	0	1	4	4	3	2	1	1	0	16	1.14 (0.58 -2.22) A
Suspect assumed positive	1	0	0	0	0	0	0	0	8	8	3	3	2	1	1	26	1.86 (0.86–4.00) A
	2	0	0	0	0	1	1	1	7	7	6	3	2	3	2	33	2.36 (1.36–4.00) B
	3	0	0	0	0	0	0	0	8	7	3	3	2	1	1	25	1.79 (0.84–3.79) A
	4	0	0	0	0	0	1	1	9	9	7	5	4	3	3	42	3.00 (1.71–5.27) B
	5	0	0	0	0	1	1	1	7	6	2	2	2	1	0	23	1.64 (0.83–3.23) A
	6	0	0	0	0	1	1	0	6	7	4	2	3	0	2	26	1.86 (0.98–3.52) A

a Misclassification was established as follows. False positive, any positive results on samples from M. flocculare, M. hyorhinis, M. hyosynoviae, or negative control groups or from the M. hyopneumoniae group < dpi 21 (no penalty incurred for suspect results on 14 or 17 dpi). False negative, any negative results on samples from the M. hyopneumoniae group ≥ dpi 21.

b ELISA 1, SK108 Mhyo, BioChek, Berkshire, UK; ELISA 2, INgezim *M. hyo* Compac, Eurofins Ingenasa, Madrid, Spain; ELISA 3, *M. hyo* Ab test, IDEXX Laboratories Inc.; ELISA 4, ID Screen Mycoplasma hyopneumoniae Indirect, IDvet, Grabels, France; ELISA 5, Civtest Suis Mhyo, Laboratorios Hipra, S.A., Girona, Spain; ELISA 6, IDEIA Mycoplasma hyopneumoniae EIA kit, Oxoid Limited, Hampshire, UK.

c Misclassification error rates calculated by Poisson regression using generalized estimating equations (GEE). Letters indicate nonsignificant (same number) or significant (different number) differences in error rates (*P *≤ 0.05; Holm-Sidak adjustment).

### Study 2.

**FIG 1:**
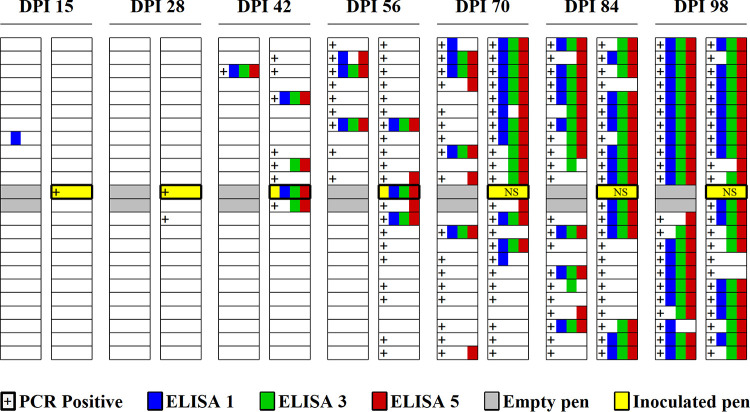
M. hyopneumoniae DNA and antibody detection by day postinoculation (dpi) in a room housing 1,250 pigs in 46 pens (23 white rectangles by 2 columns) in study 2. Ten pigs in a centrally located pen (yellow rectangle) were intratracheally administered with M. hyopneumoniae (strain 232). Thereafter, one noninoculated pig per pen was sampled/tested at each time point. M. hyopneumoniae DNA and antibody-positive results from noninoculated pigs were represented by “+” for positive PCR in tracheal samples and by squares colored by one of each the three ELISAs (ELISA 1, SK108 Mhyo, BioChek, Berkshire, UK; ELISA 3, *M. hyo* Ab test, IDEXX Laboratories Inc., Westbrook, ME, USA; ELISA 5, Civtest Suis Mhyo, Laboratorios Hipra, S.A., Girona, Spain) for positive result in serum samples. Results were based on PCR tracheal samples (15 and 28 dpi) and blood samples (15, 28, 42, and 56 dpi) collected from one noninoculated pig at each time point. In the remaining pens, tracheal and blood samples were collected from one pig at 15, 24, 42, 56, 71, 85, and 98 dpi. Serum samples with suspect classification were assumed positive. NS, no sample collected.

M. hyopneumoniae DNA testing of tracheal samples from M. hyopneumoniae-inoculated pigs (*n* = 10) confirmed productive infection, i.e., all M. hyopneumoniae-inoculated pigs were positive for M. hyopneumoniae DNA testing at 7 dpi and a noninoculated pen mate at 15 dpi. In noninoculated pens, M. hyopneumoniae DNA testing of tracheal samples verified transmission to other animals throughout the room over time, with the first positive at 28 dpi (Fig. 1).

Based on the results from study 1, ELISA serum testing results were interpreted based on the manufacturers’ cutoffs, with suspect interpreted as a positive result for IDEXX and Hipra ELISAs. Serum samples (*n* = 8) from inoculated pigs were antibody negative at 0 dpi, with the exception of one sample positive on BioChek ELISA. At 15 dpi, 5 of 10 serum samples were antibody positive on IDEXX and Hipra ELISAs, and 7 of 10 were antibody positive on BioChek. All M. hyopneumoniae-inoculated pigs were antibody positive on all the three ELISAs at 42 and 56 dpi. In the noninoculated pigs, all ELISAs were negative until 42 dpi, including pigs sharing the pen with the 10 M. hyopneumoniae-inoculated pigs, with the exception of one BioChek-positive sample at 15 dpi (Fig. 1). Overall, 318 serum test results from noninoculated pigs were used to estimate the diagnostic sensitivity and specificity of BioChek, IDEXX, and Hipra ELISAs. As shown in Table 6, the analyses found no statistically significant difference in diagnostic sensitivity, diagnostic specificity, or AUC among the 3 ELISAs.

**TABLE 6:** Diagnostic sensitivity and specificity^*a*^ of three M. hyopneumoniae antibody ELISAs in study 2

ELISA^*b*^	Sensitivity	Specificity	AUC
1	47.33 (40.0, 54.7)	99.24 (95.8, 99.8)	0.805^*a*^ (0.77, 0.84)
3	55.69 (48.9, 62.2)	98.82 (95.7, 99.7)	0.829^*a*^ (0.79, 0.86)
5	61.65 (53.8, 68.4)	98.83 (95.7, 99.7)	0.845^*a*^ (0.81, 0.87)

a Diagnostic sensitivity and specificity estimated by logistic regression using generalized estimating equations (GEE). “True status” based on detection of M. hyopneumoniae DNA in tracheal samples. Receiving operator curve and area under the curve analyses were calculated based on the manufacturer's cutoff, and suspect classification was assumed positive.

b ELISA 1, SK108 Mhyo, BioChek, Berkshire, UK; ELISA 3, *M. hyo* Ab test, IDEXX Laboratories Inc.; ELISA 5, Civtest Suis Mhyo, Laboratorios Hipra, S.A., Girona, Spain.

## DISCUSSION

The objectives of this study were to compare the performance of commercial M. hyopneumoniae serum antibody ELISAs and to evaluate cross-reactivity to other mycoplasma species under experimental conditions (study 1) and then compare the best performing assays using field samples (study 2).

Study 1 was performed with samples from cesarean-derived, colostrum-deprived (CDCD) pigs inoculated with M. flocculare, M. hyorhinis, M. hyosynoviae, or M. hyopneumoniae under experimental conditions, which were used to achieve these objectives. The use of CDCD pigs, i.e., animals raised and maintained in a highly controlled environment free of common swine pathogens (e.g., porcine circovirus type 2 [PCV2], porcine reproductive and respiratory syndrome virus [PRRSV], swine influenza A virus (SIAV), swine mycoplasmas, etc.), provided assurance that all pigs were mycoplasma free at the beginning of the study. Moreover, using colostrum-deprived (CD) pigs is a good alternative strategy to circumvent passively acquired immunity against mycoplasmas or exposure to any mycoplasma that may occur shortly after birth. Collecting and testing serum and oral fluid specimens over the course of the observation period and lung tissue recovered from pigs at euthanasia allowed for establishing the timeline and infection status of individual animals. Serum samples of a precisely known status were then used to evaluate and compare ELISA performance.

Animals in the M. hyopneumoniae group were intratracheally inoculated with using lung homogenate strain 232 (1.0 × 10^6^ CCU/ml), a well-characterized and mildly virulent isolate (23). Inoculation of pigs with lung homogenate has been extensively used to reproduce M. hyopneumoniae disease in challenge studies under controlled (13, 16) and field conditions (24, 25). Infection using pure M. hyopneumoniae culture resulted in similar gross lesions in previous challenge models (26, 27). The timeline of productive M. hyopneumoniae infection was confirmed by PCR testing of pen-based oral fluid samples (2 pigs per pen), with the first PCR-positive sample collected at 9 dpi. At euthanasia, no lung lesions were observed, but M. hyopneumoniae was confirmed in lung homogenate by PCRs in 8 of 10 pigs. Two M. hyopneumoniae-inoculated pigs were PCR negative for lung homogenate and likewise showed no detectable antibody response on any of the ELISAs. These data supported the conclusion that these animals did not become infected either through inoculation or through exposure to M. hyopneumoniae-infected animals housed in the same room. Therefore, the analyses were based on the 8 pigs demonstrated to have become infected with M. hyopneumoniae.

In other inoculation groups, PCR testing for species-specific DNA confirmed productive infection with M. hyorhinis and M. hyosynoviae, with pen-based oral fluids first positive for M. hyorhinis and M. hyosynoviae DNA by 2 and 4 dpi, respectively (15). In contrast, exposure of pigs to M. flocculare did not produce infection, as confirmed by the negative M. flocculare DNA PCR results on oral fluids (*n* = 280) and the absence of antibody in serum samples (*n* = 140) evaluated (15), which represents a limitation of this study. As previously reported, productive infection by single inoculation with M. flocculare is challenging, presumably due to inefficient colonization of the respiratory tract (28–30). Regardless, serum samples from the M. flocculare group were tested on the commercial M. hyopneumoniae serum ELISAs and the data included in the analyses, but the specific question of the cross-reactivity of M. flocculare antibodies on M. hyopneumoniae serum ELISAs could not be addressed as previously reported (31).

The M. hyopneumoniae ELISA comparisons were based on 680 serum samples collected over the course of the study (3 to 56 dpi) from 50 CDCD pigs in 5 defined mycoplasma exposure groups. Two sources of variation in M. hyopneumoniae antibody detection were apparent: pig-to-pig variability in response to exposure to M. hyopneumoniae and assay variability performance. As described by Pieters and Maes (1), M. hyopneumoniae antibody is typically detected 3 to 8 weeks after exposure but may be absent in infected animals. In the present study, M. hyopneumoniae serum antibody-positive results were observed as early as 21 and as late as 28 dpi, albeit Oxoid ELISA produced 6 suspect results (Table 4). Most typically for studies using samples of known infection status, assay performance assessment is based on receiver operating characteristic (ROC) analysis. This approach permits the calculation of diagnostic sensitivity and diagnostic specificity as a function of assay cutoff and allows for evaluations of assay performance using area under the curve (AUC) comparisons (32). In this study, ROC analysis was precluded because of the small number of M. hyopneumoniae-infected animals. A further complication was the fact that 5 of 6 ELISAs included a suspect classification. In the field, suspect is not a viable option; animals are either M. hyopneumoniae infected or not. Therefore, the analyses were performed with suspect considered positive and suspect considered negative. For these reasons, ELISA results were analyzed by comparing alternative cutoffs to the manufacturers' recommended cutoffs. After determining the optimum cutoffs, results were evaluated in terms of misclassification error rates. Alternative assay cutoffs were calculated as the 97.5% and 99.7% quantiles of the normal distribution (upper tail values) for 5 ELISAs (BioChek, Eurofins Ingenasa, IDEXX, IDvet, and Hipra) and 2.5% and 0.3% quantiles (lower tail values) for Oxoid ELISA, as described in reference 21. A comparison of alternative cutoffs to the manufacturers’ recommended cutoffs in terms of false-positive results showed that the manufacturers’ recommended cutoffs were usually more stringent, i.e., produced fewer false positives for both the suspect considered positive and suspect considered negative conditions. Exceptions to this general observation were IDvet and Oxoid ELISAs (Table 3).

Thereafter, the M. hyopneumoniae ELISAs were analyzed in terms of misclassification error rate based on the manufacturers' cutoffs. The misclassification error rate was calculated as the total number of false negatives and false positives among samples tested for both suspect conditions, i.e., as positive or as negative (Table 5). False positive was defined as a positive result on samples from M. flocculare, M. hyorhinis, M. hyosynoviae, and negative control groups. A false negative was defined as a negative result from the M. hyopneumoniae group at ≥21 dpi. Thus, misclassification error rate simultaneously accounted for both types of diagnostic errors that occur in routine testing. Misclassification errors (false positives or false negatives) were observed in all assays evaluated. No significant difference in misclassification error rate was observed among BioChek, IDEXX, Hipra, and Oxoid ELISAs (Table 5). Among these four, the performance of Oxoid ELISA differed both in the early onset of detection and in the number of false positives in samples from non-M. hyopneumoniae-inoculated pigs.

In study 2, the three ELISAs (BioChek, IDEXX, and Hipra) that provided the highest performance in study 1 were evaluated under field conditions. Specifically, M. hyopneumoniae infection was established in a commercial wean-to-finish population (1,250 pigs in 46 pens in one room) free of M. hyopneumoniae, porcine reproductive and respiratory syndrome virus, and influenza A virus infection by intratracheal inoculation of 10 pigs with M. hyopneumoniae. Thereafter, the collection and DNA testing of tracheal samples from all pens over time was used to establish the M. hyopneumoniae infection status of individual animals and compare serum ELISAs.

The control and/or elimination of M. hyopneumoniae from commercial production systems requires ongoing testing to establish the true status of populations and detect the introduction of the pathogen. In study 1 (CDCD pigs), the three ELISAs (BioChek, IDEXX, and Hipra) with the best performance were equivalent when comparing diagnostic specificity and false-positive rates. Notably, IDEXX and Hipra performed best by interpreting suspect results as positive. Likewise, in study 2, although the BioChek ELISA produced 2 false-positive results based on DNA testing, no statistically significant differences were detected in the diagnostic sensitivity or specificity of BioChek, IDEXX, and Hipra ELISAs. The point of conflict in the monitoring process has been (and continues to be) maximizing early detection while minimizing false-positive reactions. Future research should focus on improving diagnostic methods in order to be able to improve time to detection and overall diagnostic sensitivity. For the present, the data reported in this study will help users understand ELISA performance and select the assay (or combination of assays) most suited to their testing objective(s).
